# Development of a multiplex quantitative real-time PCR (qPCR) assay for simultaneous detection of *Schistosoma haematobium* and high-risk human papillomaviruses

**DOI:** 10.1371/journal.pntd.0014694

**Published:** 2026-09-11

**Authors:** Emmanuel Timmy Donkoh, Isaac Williams, Rita Nyaaba Akologo, Alfred Afriyie Asiedu, Reindoff Junior Ofori-Atta, Ivy Wina Boadu, Oksana Ryabinina, Akua Obeng Forson, Kwadwo Boampong, Neeraj Jain, Edward Tieru Dassah, Samuel Fosu Gyasi, Laila Sara Arroyo Mühr, William Gariba Akanwariwaik

**Affiliations:** 1 Centre for Research in Applied Biology, University of Energy and Natural Resources, Sunyani, Ghana; 2 Department of Medical Diagnostics, Faculty of Allied Health Science, Kwame Nkrumah University of Science and Technology, Kumasi, Ghana; 3 Department of Chemical Pathology, School of Medical Sciences, University of Cape Coast, Cape Coast, Ghana; 4 Department of Medical Laboratory Science, University of Ghana, Accra, Legon, Ghana; 5 Department of Theoretical and Applied Biology, Kwame Nkrumah University of Science and Technology, Kumasi, Ghana; 6 Dr. K C Patel Research and Development Centre (KRADLE), Charotar University of Science and Technology (CHARUSAT), Changa, India; 7 Department of Population, Family and Reproductive Health, School of Public Health, Kwame Nkrumah University of Science and Technology, Kumasi, Ghana; 8 Department of Biological Science, University of Energy and Natural Resources, Sunyani, Ghana; 9 International HPV Reference Center, Center for Cervical Cancer Elimination, Karolinska Institutet, Stockholm, Sweden; National Institutes of Allergy and Infectious Diseases, NIH, UNITED STATES OF AMERICA

## Abstract

**Background:**

Female genital schistosomiasis (FGS) and high-risk human papillomavirus (hr-HPV) infections frequently co-occur in sub-Saharan Africa and may jointly contribute to cervical carcinogenesis. However, no existing molecular platform enables simultaneous detection of *Schistosoma haematobium* and hr-HPV from a single cervical specimen.

**Methods:**

A multiplex quantitative real-time PCR (qPCR) assay targeting the *S. haematobium* Dra1 repeat and seven hr-HPV genotypes (16, 18, 31, 33, 45, 52, 58) was developed and validated. Analytical performance was assessed using HPV reference plasmids from the International HPV Reference Centre and biobanked *S. haematobium* DNA from the Centre for Research in Applied Biology (CeRAB), Ghana. Clinical performance was evaluated using 217 archived cervical swabs from high-risk women enrolled in the CERVIVAL project in rural Ghana.

**Results:**

The assay showed a limit of detection of 10 copies/µL for all targets, with no cross-reactivity to non-target HPV genotypes, common uropathogenic bacteria, or *Schistosoma mansoni*. In reference panels, positive and negative agreement were 98.2% and 100% for *S. haematobium* and 100% for both sensitivity and specificity across all hr-HPV genotypes. In the 217 high-risk cervical samples, *S. haematobium* DNA was detected in 56.7% (123/217). HPV58 (19.8%), HPV16 (14.7%), and HPV52 (12.4%) were the most frequent genotypes. Concordance with visual inspection/microscopy for FGS was 93.6% (κ = 0.87), and concordance with a molecular HPV test (ScreenFire HPV) ranged from 90.3% to 99.5% (κ = 0.80–0.95) across HPV channels. The assay identified 11 additional *S. haematobium*–positive samples that were negative by visual inspection/microscopy.

**Conclusions:**

The multiplex qPCR assay provides a highly sensitive and practical tool for integrated detection of *S. haematobium* and hr-HPV from a single cervical sample. Its compatibility with standard qPCR platforms and strong agreement with established diagnostic methods support its potential use in FGS and cervical cancer screening programmes in resource-limited, co-endemic settings.

## Author summary

In sub-Saharan Africa, millions of women face the dual threat of infections that severely impact reproductive health: a parasitic worm known as *Schistosoma haematobium*, which causes female genital schistosomiasis, and high-risk types of human papillomavirus, the primary cause of cervical cancer. Growing evidence suggests that suffering from both infections simultaneously may increase a woman’s risk of developing cervical cancer. However, health programs currently lack a simple method to test for both pathogens using a single clinical sample. To address this gap, we developed a new laboratory test capable of detecting DNA from both the parasite and the virus in a single cervical swab. When we evaluated this tool using samples from women in Ghana, we found it to be highly accurate. By combining these diagnoses into one highly sensitive tool that uses standard laboratory equipment, we can streamline screening programs. This approach reduces testing resources and could potentially allow health workers to identify and treat these conditions earlier, offering a practical solution to help prevent cervical cancer in regions where both diseases are widespread.

## Introduction

Female genital schistosomiasis (FGS) is a chronic gynaecological condition caused by infection with *Schistosoma haematobium*, in which parasite eggs become deposited in the cervix, vagina, and other pelvic tissues [1]. Their presence triggers persistent granulomatous inflammation, epithelial disruption, and characteristic mucosal alterations such as sandy patches and rubbery papules. Although FGS affects millions of women in endemic regions, it remains markedly underdiagnosed [2,3]. Its clinical signs are subtle, highly variable, and often indistinguishable from sexually transmitted infections, leading to both missed diagnoses and delays in treatment [2,4]. In many resource-limited settings, the true burden of FGS is therefore underestimated [5].

In recent years, increasing attention has been given to the interaction between FGS and infections with high-risk human papillomavirus (hr-HPV), the causal agent of cervical cancer. Chronic inflammation and mucosal damage associated with *S. haematobium* infection may facilitate the acquisition and persistence of hr-HPV, both of which are key determinants of cervical carcinogenesis [6,7]. Several epidemiological and clinical studies suggest that women with concurrent FGS and hr-HPV infections may have a heightened risk of cervical precancerous lesions [6,8]. This concern is particularly relevant in sub-Saharan Africa, where both conditions are highly prevalent and frequently affect women during their reproductive years [7,9]. Globally, hr-HPV genotypes, including HPV16, 18, 31, 33, 35, 45, 52, and 58, account for the vast majority of cervical cancer cases [10], yet genotype distribution differs by region, underscoring the importance of context-specific diagnostic strategies [11].

Despite this recognised overlap, reliable detection of both infections remains challenging. Diagnosis of FGS continues to rely mainly on visual inspection of the cervix and urine microscopy [12,13]. Both methods have modest sensitivity, especially in cases of low-intensity infection or when egg shedding is intermittent [12,13]. Microscopy often fails to identify early or subclinical disease, while visual inspection is operator-dependent and can miss characteristic lesions, particularly in older women, where the transformation zone becomes less visible [12,14]. In the case of HPV, commercial molecular assays are widely used for primary cervical screening, but they require specialised laboratory infrastructure and are costly to implement at scale in rural or underserved areas [15,16]. The multiplex assay similarly requires such infrastructure; however, its ability to detect multiple targets simultaneously may improve testing efficiency and has the potential to reduce overall costs.

Importantly, no existing diagnostic platform is capable of detecting FGS and hr-HPV simultaneously from the same cervical specimen. This represents a major gap in settings where integrated screening would be both clinically relevant and programmatically efficient. The World Health Organisation has emphasised the need for coordinated approaches that address cervical cancer prevention and neglected tropical diseases together, particularly in regions where they overlap [17,18]. A single, sensitive molecular assay capable of detecting *S. haematobium* and hr-HPV could support such integrated models, reduce costs, minimise patient visits, and improve early diagnosis.

Multiplex quantitative real-time PCR (qPCR) provides an opportunity to address this gap. Real-time PCR assays targeting the *S. haematobium* Dra1 sequence have shown markedly higher sensitivity than microscopy, while type-specific PCR for hr-HPV genotypes is the established benchmark for accurate viral detection [19,20]. However, to date, no validated assay has combined these targets within a single multiplex reaction suitable for routine clinical use in low-resource environments.

This study developed and validated a qPCR assay designed to detect the *S. haematobium* Dra1 repeat and the seven most clinically relevant hr-HPV genotypes (16, 18, 31, 33, 45, 52, and 58) using a single cervical sample. Analytical sensitivity, specificity, and clinical performance were evaluated through reference materials and cervical swabs collected from women in rural Ghana, a population with a high likelihood of co-infection. The goal of this work was to provide a sensitive, specific, and accessible molecular tool that can support early diagnosis and integrated screening programs in schistosomiasis- and HPV-endemic regions.

## Methods

**Fig 1:**
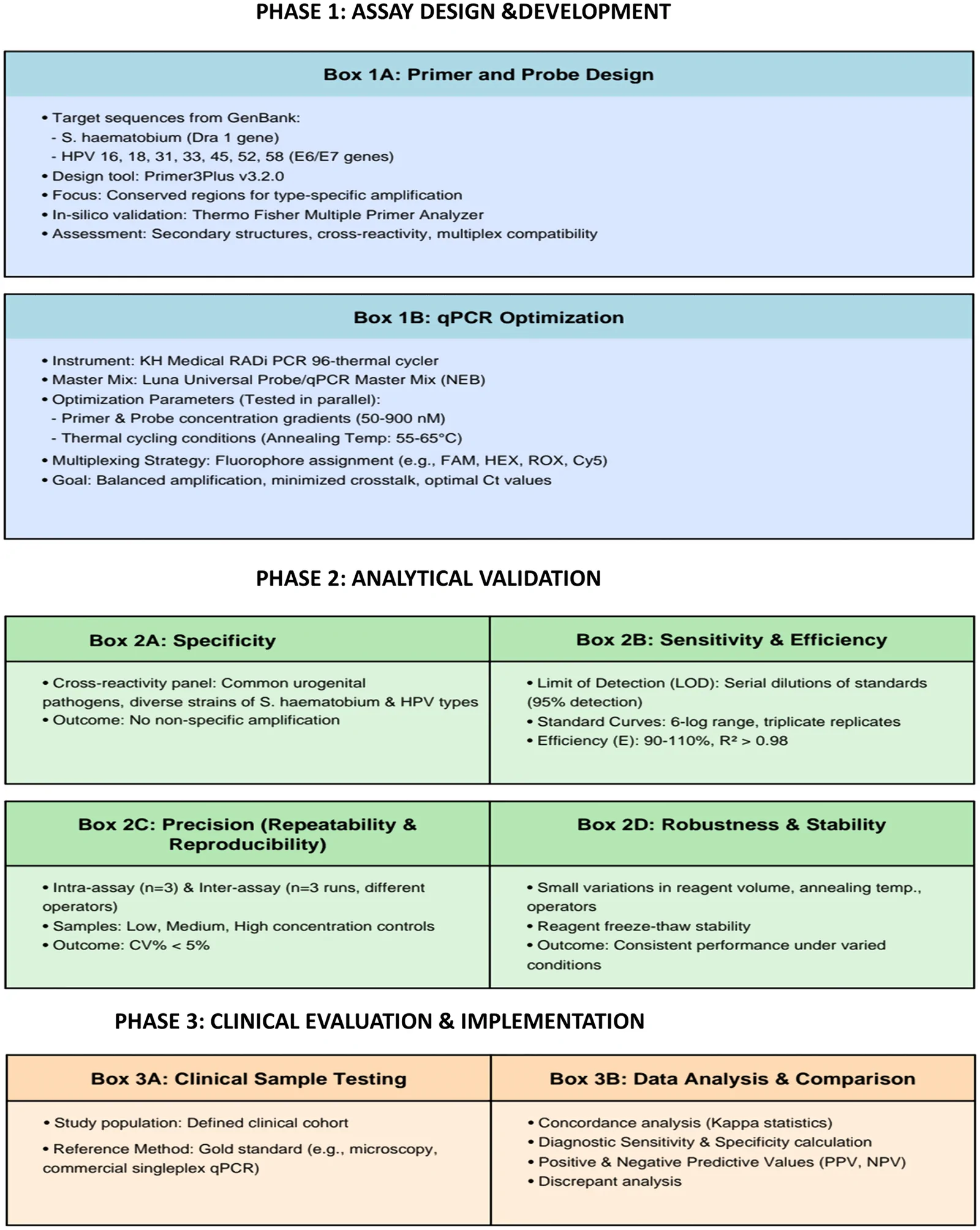
Workflow for multiplex quantitative real-time PCR (qPCR) assay development.

This study involved the development, optimization, analytical validation, reference sample performance and clinical evaluation of a qPCR assay capable of detecting *Schistosoma haematobium* and seven high-risk human papillomavirus (hr-HPV) genotypes (HPV16, 18, 31, 33, 45, 52, and 58) from cervical samples. The methodological workflow, shown in Fig 1, included primer and probe design, in-silico evaluation, analytical specificity and sensitivity testing, and validation using reference materials and clinical specimens collected in Ghana.

### Primer and probe design

To design a multiplex assay compatible with simultaneous detection of parasitic and viral targets, DNA sequences for the *S. haematobium* Dra1 repeat region and the *E6/E7* genes of the seven hr-HPV types were obtained from GenBank. Primers and TaqMan probes were designed using Primer3Plus version 3.2.0 [21], focusing on conserved regions that enable type-specific amplification. All oligonucleotides were assessed in silico for secondary structures, cross-reactivity, and multiplex compatibility using the Thermo Fisher Multiple Primer Analyzer [22]. Primers and probes were synthesised by Dynegene Biotech Company Limited (Dynegene, China) and are shown in Table 1.

**Table 1:** Primers and probes targeting the E6/E7 region of the HPV genome and Dra1 repeated sequence of S. haematobium.

Target	Gene	Primers and Probe (3’ to 5’)	Reporter	Quencher	Length	Accession Number
HPV16	E6/E7	F: ATAATGGACTGGTGGGCGTAC R: TGCTGATAGGTCGCTGGTTC P: GCGCCGCCCCACCAACCGGT	ROX	BHQ-2	128 bp	FJ229356.1
HPV18	E6/E7	F: CGTGGCGTCCGTGGAATAAT R: TGCTGTCCTTGCTGAGGTTG P: GCCAACGTCGTGCTTACCGTGACCGGA	HEX	BHQ-1	124 bp	KJ869426.1
HPV 31	E6/E7	F: GAACCGAAAACGGTTGGTATATA R: ATCGTAGGGTATTTCCAATGC P: CATAGTATTTTGTGCAAACCTACAGACGCCATGT	CY5	BHQ-2	144 bp	KJ869427.1
HPV 45	E6/E7	F: CAGTGTAATACATGTTGTGACCAG R: ACAGGATCTAATTCATTCTGAGGT P: CAAGAAAGACTTCGCAGACGTAGGGAAACAC	CY5	BHQ-2	88 bp	PP818823.1
HPV 33	E6/E7	F: GCATGATTTGTGCCAAGCAT R: CTCAGATCGTTGCAAAGGTTT P: ACTATACACAACATTGAACTACAGTGCGTGGAATGC	CY5	BHQ-2	148 bp	KJ869428.1
HPV 52	E6/E7	F: AAACGGTCAGACCGAAACC R: CAGCACCTCACACAATTCGT P: AACACAGTGTAGCTAACGCACGGCCATGT	CY5	BHQ-2	116 bp	KJ869430.1
HPV 58	E6/E7	F: CACGGACATTGCATGATTTGT R: TCAGATCGCTGCAAAGTCTTT P: TTTCAATTCGATTTCATGCAC	CY5	BHQ-2	97 bp	KJ869432.1
*S. haematobium*	Dra 1	ShF: GATCTCACCTATCAGACGAAAC ShR: TCACAACGATACGACCAAC ShP: TGTTGGTGGAAGTGCCTGTTTCGCAA	FAM	BHQ1–3	121 bp	XM_051209008.1

Note: F- Forward primer; R-Reverse primer; P- TaqMan probe, BHQ-1 (Black Hole Quencher 1), BHQ-2 (Black Hole Quencher 2); BHQ-3 (Black Hole Quencher 3).

### Validation panels and composition

Validation panels used for assay validation consisted of four distinct sample sets (Table A in S1 Appendix). First, HPV DNA-containing plasmids (HPV16, 18, 31, 33, 45, 52, and 58) obtained from the International HPV Reference Center (IHRC) were used, together with biobanked *S. haematobium* DNA (Analytical Reference Panel (ARP)), for analytical validation, including assessment of primer-probe performance, analytical specificity, and limit of detection. Secondly, to assess the clinical performance of the *S. haematobium* component, DNA was extracted from a clinical panel of 94 urine samples (56 microscopy-positive and 38 microscopy-negative) obtained from a previous parasitological study conducted at CeRAB in Ghana (*S. haematobium* Clinical Reference Panel (SCRP)) [23,24]. These samples served as well-characterized material for evaluating the clinical performance of the Dra1 assay. Third, to evaluate the clinical performance of the HPV component, 132 previously extracted cervical DNA samples (HPV Clinical Reference Panel (HCRP); 87 HPV-positive and 45 HPV-negative) were included; all had been independently genotyped using the commercial ScreenFire HPV assay and therefore provided genotype-confirmed reference material for the seven high-risk HPV types.

### Clinical validation panel and study population

Multiplex qPCR optimisation and clinical validation were conducted on archived cervical swabs (CERVIVAL Clinical Validation Panel (CCVP; n = 217)) from women enrolled in the CERVIVAL (Implementation of self-sampling strategy to increase cervical cancer and female genital schistosomiasis screening) project at the Centre for Research in Applied Biology (CeRAB), Sunyani, Ghana, a combined cervical cancer and schistosomiasis screening initiative [23]. All women enrolled in the CERVIVAL study underwent visual inspection with acetic acid (VIA) for cervical assessment, urine microscopy for detection of *Schistosoma haematobium* ova, and completion of a structured symptom questionnaire. Cervical examinations were performed by trained gynaecologists experienced in cervical cancer screening and FGS identification, following standardized protocols. Risk assessment incorporated findings from cervical inspection for FGS-like lesions, parasitological confirmation of *S. haematobium*, and reported symptoms including water exposure, urinary discomfort, genital symptoms, and abnormal bleeding. Among the participating women (n = 1,625), individuals with a cumulative risk score >6 (n = 217) were classified as high risk, and all participants meeting this criterion were included in the present study. These cervical swabs had been previously analyzed using the ScreenFire HPV assay, and the resulting genotypes were used as the reference for HPV validation (reproducibility testing)

As this study utilized archived, well-characterized biobank samples, no a priori sample size calculation was performed; however, the available sample sizes were deemed sufficient for evaluation of diagnostic performance and reproducibility.

### Sample preparation and DNA extraction

Frozen cervical swabs were thawed and incubated overnight in 500 µL phosphate-buffered saline to maximize epithelial cell release. Samples were vortexed and centrifuged to obtain cellular pellets, which were washed and processed for DNA extraction using the Zymo Research Quick-DNA Miniprep Plus Kit, following the manufacturer’s instructions. DNA was purified following the manufacturer’s recommended protocol, eluted in 50 μL, and quantified using a Qubit 4 Fluorometer with the Qubit dsDNA High Sensitivity (HS) Assay.

### Quantitative PCR optimization and amplification conditions

Quantitative PCR optimization for each individual target (single-plex) was performed in triplicate using a KH Medical RADI PCR 96 thermal cycler (KH Medical, Korea). Three primer-probe configurations were evaluated to identify the concentrations yielding the strongest and most consistent amplification. Each 20 μL reaction contained 10 μL of Luna Universal Probe PCR Master Mix (New England Biolabs, USA), 5 µL of DNA template (22 ng/µL), and nuclease-free water. Biobanked *S. haematobium* DNA and HPV reference plasmids from the International HPV Reference Center (IHRC), representing genotypes HPV16, 18, 31, 33, 45, 52, and 58, were used as templates.

The first configuration used 0.8 μL of each primer and 0.4 μL of probe (10 μM); the second used 0.5 μL of each primer and 0.5 μL of probe; and the third used 1 μL of each primer with 0.5 μL of probe. Amplicon specificity and absence of primer–dimer formation were confirmed by gel electrophoresis. To determine the optimal annealing temperature, a gradient from 58°C to 62°C was tested. The final cycling protocol included 95°C for 60 seconds, followed by 40 cycles of 95°C for 15 seconds and 60°C for 30 seconds, with fluorescence acquisition at the annealing/extension step.

### Analytical specificity

The analytical specificity of the developed qPCR assay was assessed by testing a panel of non-target organisms and closely related genomes: for bacterial DNA (*Escherichia coli, Klebsiella pneumoniae, Staphylococcus aureus*), non-target HPV genotypes (HPV16 tested with HPV18 assay, HPV18 tested with HPV 45 assay), and DNA from the related parasite *Schistosoma mansoni*. Analytical specificity was assessed in single-plex format to evaluate each target individually. Testing included (i) Biobanked reference DNA of *S. haematobium* (ARP) was retrieved from CeRAB, Ghana and (ii) HPV reference plasmids from the IHRC representing each target genotype (HPV 16, 18, 31, 45, and 58) (ARP) in a single-plex reaction (individual targets). All reactions were run in triplicate together with negative controls (nuclease free water) and non-template controls (NTCs). Specificity was defined as the complete absence of amplification (Ct > 40 or undetectable) in negative controls (nuclease free water) and NTCs, and exclusive amplification of the intended target in positive controls.

### Analytical sensitivity and limit of detection

Ten-fold serial dilutions of reference DNA (positive controls) (from 10^7^ to 10^0^ copies/μL) were prepared for *S. haematobium* and each hr-HPV genotype (Equation 1). Each dilution was amplified in 20 replicates in multi-plex format to determine the performance of each primer-probe set. The limit of detection (LoD) was defined as the lowest concentration at which ≥95% of replicates were positive. Amplification efficiency was calculated from standard curves generated using Ct values across dilution series as described by [25].


$$\begin{array}{c} Copy\;number=\frac{X(ng)\;x\;(\text{6}.\text{0}\text{2}\text{2}\text{1}\;x{\text{1}\text{0}}^{\text{2}\text{3}})}{\text{N }\times \text{ 660}(\text{g}/\text{mol})\text{x }({\text{10}}^{\text{9}}\;(\text{ ng}/\text{g})\;} \end{array}$$ (1)

X (ng) = Amount of amplicon

660 (g/mol) = Average mass of 1bp dsDNA

N = Length of dsDNA amplicon

6.0221 × 10^23^ = Avogadro′ s constant

10^9^ (ng/g) = Conversion factor

### Real-world clinical validation panel

Clinical performance was first assessed in a single-plex qPCR format using known-status samples. For this, SCRP comprising DNA from a clinical panel of 94 urine samples (56 positive and 38 negative) obtained from a previous parasitological study were used to assess the performance of the Dra1 component. For HPV, 132 extracted cervical DNA samples previously characterized with the ScreenFire commercial HPV assay (HCRP) were selected. This included 87 HPV-positive samples, with at least four samples representing each of the seven most common hr-HPV genotypes.

### Transition to multiplex qPCR format

Following single-plex optimization, all primer-probe sets were combined to establish the multiplex format, a process that required careful optimization to balance amplification across targets. Optimization involved adjusting primer and probe concentrations, testing fluorophore channel compatibility, and allocating targets across two parallel reactions to minimize spectral overlap and competition. Multiplex performance was evaluated by comparing cycle threshold (Ct) values obtained in single-plex versus multiplex reactions. Differences in Ct values were monitored for each target, and primer-probe concentrations were adjusted to maintain Ct variation within ±1 cycle, which was considered acceptable for reliable detection. The primer and probe concentrations for each target in the multiplex assay are provided in the supplementary file (S1 File)

### Evaluation in 217 cervical swabs

The optimized multiplex qPCR assay was applied to CCVP. To minimize probe competition and fluorophore spectral overlap, targets were distributed across two parallel reactions: Reaction 1 amplified *S. haematobium* Dra1, HPV16, HPV18, and HPV45, while Reaction 2 pooled HPV31, HPV33, HPV52, and HPV58 into a single fluorophore channel. In the pooled channel, the assay generated a positive signal if any of the four HPV targets were present; positive samples were subsequently resolved using single-plex reactions. Each 20 µL reaction contained 10 µL of Luna Universal Probe PCR Master Mix, 5 µL of DNA template, and 0.5 µL of each primer and probe (10 µM). Cycling conditions followed the previously optimized protocol. Ct values, detection frequencies, and inter-run reproducibility were recorded for all samples. Assay results were compared with visual inspection for FGS-like lesions and urine microscopy for *S. haematobium* infection, and with the ScreenFire assay for high-risk HPV genotyping.

### Reproducibility of the novel assay

Clinical evaluation data from visual inspection of the cervix (for FGS-associated signs) and urine microscopy (for *S. haematobium* ova) were used as the reference standard (CCVP) to assess the performance of the developed multiplex assay. For HPV genotyping, the 217 preselected archived cervical swabs had been previously analysed using the commercial ScreenFire HPV assay, which served as the reference standard for evaluating multiplex assay reproducibility.

### Multiplex qPCR agreement analysis and statistics

Agreement between the different diagnostic methods was assessed using overall concordance, positive and negative concordance, positive predictive value (PPV), negative predictive value (NPV), and Cohen’s kappa (κ). Cohen’s kappa was used to quantify agreement between the developed multiplex assay and the reference standard, with values interpreted according to established thresholds: 0.0-0.2 indicating poor or slight agreement, 0.2-0.4 fair agreement, 0.4-0.6 moderate agreement, 0.6-0.8 substantial agreement, and 0.8–1.0 almost perfect agreement. Associations between diagnostic outcomes were examined using chi-square tests. All statistical analyses, including concordance, positive and negative predictive values, and Cohen’s kappa, were performed using IBM SPSS Statistics version 29 (IBM Corp., Armonk, NY, USA).

### Ethical considerations

This study used fully anonymized archived samples collected under prior ethical approval from the Committee for Human Research and Ethics at the University of Energy and Natural Resources (CHRE/AP/108/023). Only secondary analyses were performed, and no new participants were recruited. All procedures complied with institutional ethical guidelines for the use of archived human research materials.

## Results

### Primer-probe performance and analytical sensitivity/specificity

All primer-probe sets demonstrated consistent amplification of their intended targets in single-plex qPCR format, with Ct values < 30 for all positive controls. Mean Ct values were 17.7 ± 0.4 for *S. haematobium* and 21.6-27.5 across the HPV plasmids (Table 2), indicating efficient amplification. No amplification was observed in negative controls or NTCs, confirming absence of contamination and good assay performance.

**Table 2:** Cycle threshold values observed in the single-plex qPCR assay positive and negative controls.

Target	Gene	Sample type	Mean Ct value observed
*S. haematobium*	Dra 1	Positive control	17.7 ± 0.4
*S. haematobium*	Dra1	Negative control	Undetected
HPV 18	E6/E7	Positive control	23.6 ± 0.4
HPV 16	E6/E7	Positive control	21.6 ± 0.4
HPV 45	E6/E7	Positive control	28.1 ± 0.2
HPV 31	E6/E7	Positive control	27.5 ± 0.2
HPV 58	E6/E7	Positive control	24.5 ± 0.5
HPV	E6/E7	Negative control	Undetected

### Analytical specificity

Analytical specificity was evaluated in single-plex qPCR format using a panel of non-target organisms and closely related genomes (Table B in S1 Appendix). No amplification was observed for bacterial DNA (*Escherichia coli, Klebsiella pneumoniae, Staphylococcus aureus*), non-target HPV genotypes (e.g., HPV16 tested with the HPV18 assay; HPV18 tested with the HPV45 assay), or DNA from the related parasite *Schistosoma mansoni*, with no Ct values detected within 40 amplification cycles. These results demonstrate high target specificity and absence of cross-reactivity

### Analytical sensitivity and limit of detection

Ten-fold serial dilutions of ARP positive-control DNA (10^7^ -10^0^copies/µL) were tested for *S. haematobium* and each hr-HPV genotype. The lowest concentration at which ≥95% of replicates yielded positive amplification was 10 copies/µL for all targets, establishing the limit of detection (LoD) (Table C in S1 Appendix). Standard curves demonstrated acceptable amplification efficiencies (90–110%), confirming high analytical sensitivity across all primer-probe sets (Figs A, B, and C in S2 Appendix).

### Clinical performance in reference samples

Clinical performance of the assay was first assessed in a single-plex qPCR format using SCRP (Table 3). In the *S. haematobium* Clinical Reference Panel (SCRP; n = 94), the multiplex qPCR assay showed positive agreement of 98.2% (55/56) and negative agreement of 100% (38/38) with prior urine microscopy results. For evaluation of the hr-HPV component, HCRP DNA samples were analysed. This panel included 87 hr-HPV-positive and 45 negative specimens, representing all seven targeted genotypes. The multiplex qPCR assay detected all 87 positive samples and all 45 negatives, achieving 100% clinical sensitivity and specificity for each of the seven hr-HPV types.

**Table 3:** Clinical performance in reference samples.

Target	Clinical sensitivity % (95% CI)	Clinical specificity % (95% CI)	PPV % (95% CI)	NPV % (95% CI)
*S. haematobium*	98.2 (90.7–99.9)	100.0 (90.8–100.0)	100.0 (93.5–100.0)	97.4 (86.2–99.5)
hr-HPV	100.0 (95.8–100.0)	100.0 (92.1–100.0)	100.0 (95.8–100.0)	100.0 (92.1–100.0)

### Performance evaluation in clinical validation panel

To evaluate the performance of the developed assay in a clinical setting, 217 high-risk cervical samples from the CERVIVAL cohort (CCVP) were analyzed in separate single-plex qPCR reactions (Table D in S1 Appendix). The assay detected the Dra1 gene of *S. haematobium* in 123/217 (56.7%) samples, with Ct values ranging from 15.7 to 34.8 (mean Ct: 27.1). Likewise, a separate reaction was performed for the seven most common high-risk HPV genotypes (Table D in S1 Appendix). HPV 58 was the most common (43/217,19.8%) detected genotype, followed by HPV 16 (32/217, 14.7%), HPV 52 (27/217, 12.4%), and HPV 33 (21/ 217, 9.7%), with mean Ct values ranging from 24.5 to 25.4. A low positivity rate was observed for HPV 18 (19/217, 8.8%), HPV 31 (14/217, 6.5%), and HPV 45 (9/217, 4.1%), with mean Ct values ranging from 25.2 to 26.1.

The validated single-plex qPCR conditions were applied to the multiplex qPCR assay. The results obtained in the developed multiplex assay were consistent with those observed in the single-plex reaction. The Ct values obtained from the multiplex qPCR assay are presented in Table 4. The Dra1 gene was detected in 123/217 (56.7%) of the samples with Ct values ranging from 16.9 to 34.0 and a mean of 26.9 ± 5.6**.** Among the HPV genotypes, HPV 58 was the most common (43/217, 19.8%) detected genotype with Ct values ranging from 20.2 to 31.1, and a mean value of 24.7 ± 3.1, followed by HPV 16 (32/217, 14.7%**,** mean Ct: 25.2 ± 3.3). Moderate detection rates were observed for HPV 33 (21/217, 9.7%), HPV 18 (19/217, 8.8%), and HPV 31 (14/217, 6.5%), while HPV 45 had the lowest prevalence rate of 5.7% (11/217) with a mean Ct of 25.5 ± 2.5.

**Table 4:** Cycle threshold values observed in the multiplex qPCR performance for 217 clinical samples.

Target Gene	n (%)	Ct Min	Ct Max	Mean Ct ± SD
*S. haematobium* (Dra 1Gene)	123(56.7)	16.8	34.0	26.9 ± 5.6
HPV 16(E6/E7)	32(14.7)	20.2	30.9	25.2 ± 3.3
HPV 18(E6/E7)	19(8.8)	20.1	30.1	25.4 ± 2.7
HPV 45(E6/E7)	11(5.7)	21.8	30.1	25.5 ± 2.5
HPV 31(E6/E7)	14(6.5)	22.7	30.0	25.7 ± 2.1
HPV 33(E6/E7)	21(9.7)	20.1	30.1	24.4 ± 3.5
HPV 52(E6/E7)	27(12.4)	20.2	31.4	24.9 ± 3.4
HPV 58(E6/E7)	43(19.8)	20.2	31.1	24.6 ± 3.1

N: the number of positive cases.

%: the percentage of positive cases.

### Agreement with standard diagnostic methods

Agreement between the multiplex qPCR assay and routine diagnostic methods was assessed. For S. haematobium, comparison with visual inspection of the cervix and urine microscopy identified 115 positive cases. The multiplex PCR assay detected 112 of these 115 cases, and additionally identified 11 further Dra1-positive samples that were negative on visual inspection/microscopy (Table 5).

**Table 5:** Comparative analysis of the developed qPCR performance against the standard assay.

Target		VI/Microscopy Negative	VI/Microscopy Positive	Total
***S. haematobium***	qPCR Negative	91	3	94
	qPCR Positive	11	112	123
	Total	102	115	217
		ScreenFire Negative	ScreenFire Positive	Total
**HPV 16**	qPCR Negative	184	1	185
	qPCR Positive	3	29	32
	Total	187	30	217
**HPV 18**	qPCR Negative	198	0	198
	qPCR Positive	2	17	19
	Total	200	17	217
**HPV 45**	qPCR Negative	205	1	206
	qPCR Positive	0	11	11
	Total	205	12	217
**HPV31,33,52,58**	qPCR Negative	128	**2**	130
	qPCR Positive	18	69	87
	Total	146	71	217

VI = visual inspection of the cervix; qPCR: quantitative polymerase chain reaction PCR.

For hr-HPV, results obtained by the multiplex qPCR assay were compared with ScreenFire results available for all 217 women. For HPV16, the qPCR assay detected 29 of 30 ScreenFire-positive cases and identified three additional positives that were negative by ScreenFire. For HPV18, all 17 ScreenFire-positive cases were detected and two additional positives were identified. For HPV45, the assay detected 11 of 12 positives. For the pooled HPV31/33/52/58 channel, the assay detected 69 of 71 ScreenFire-positive cases. Full results are shown in Table 5.

The multiplex qPCR assay demonstrated an overall concordance rate of 93.6% (95% CI: 89.5–96.1) with visual inspection/microscopy for detecting *S. haematobium*, with a positive concordance of 97.4% (92.6–99.1) and a negative concordance of 89.2% (81.7–93.9) (Table 6). A significant association between the methods was observed (χ² = 165.13, p < 0.001), with a Cohen’s kappa value of 0.87, indicating almost perfect agreement. The qPCR assay detected 11 additional Dra1-positive samples that were negative by visual inspection/microscopy, consistent with literature demonstrating higher sensitivity of molecular methods for sub-patent *S. haematobium* infections.

**Table 6:** Diagnostic agreement between the developed qPCR assay and the standard assay.

Parameter	*S. haematobium* (VI/microscopy)	HPV16	HPV18	HPV45	HPV31/33/52/58
Overall concordance	93.5% (89.5–96.1)	98.2% (95.4–99.3)	99.1% (96.7–99.8)	99.5% (97.3–99.9)	90.8% (85.4–93.7)
Positive concordance	97.4% (92.6–99.1)	96.7% (83.3–99.4)	100.0% (81.6–100.0)	91.7% (62.5–98.5)	97.2% (90.2–99.2)
Negative concordance	89.2% (81.7–93.9)	98.4% (95.4–99.5)	99.0% (96.2–99.7)	100.0% (98.2–100.0)	87.7% (80.5–92.4)
PPV	91.1% (84.7–94.9)	90.6% (75.8–96.8)	89.5% (66.5–97.3)	100.0% (74.1–100.0)	79.3% (69.2–86.7)
NPV	96.8% (91.0–98.9)	99.5% (97.0–99.9)	100.0% (98.0–100.0)	99.5% (97.0–99.9)	98.5% (97.0–99.9)
Cohen’s κ	0.87	0.93	0.94	0.95	0.80
p values	<0.001	<0.001	<0.001	<0.001	<0.001

PPV: Positive predictive value; NPV: Negative predictive value.

For hr-HPV, the multiplex qPCR assay also showed high concordance with the ScreenFire assay across all genotype channels. For HPV16, the overall concordance was 98.2% (95% CI: 95.4–99.3), with a negative concordance of 98.4% (95.4–99.5) and a negative predictive value of 99.5% (97.0–99.9). HPV45 showed the highest concordance at 99.5% (95% CI: 97.3–99.9), with an almost perfect level of agreement (κ = 0.95). HPV18 demonstrated an overall concordance rate of 99.1% (95% CI: 96.7–99.8). For the pooled HPV31/33/52/58 channel, the overall concordance was 90.3% (95% CI: 85.4–93.7), with substantial agreement (κ = 0.80). Significant associations between the assays were observed for all genotype categories (all p values < 0.001) (Table 6).

## Discussion

This study developed and validated a multiplex qPCR assay that allows rapid detection of the Dra1 repeated sequence of *Schistosoma haematobium* and seven common hr-HPV genotypes (16, 18, 31, 33, 45, 52, 58). By integrating parasitic and viral targets into a single reaction, the assay reduces diagnostic workload and addresses a critical gap in resource-limited settings (Table G in S1 Appendix). The assay demonstrated a limit of detection of 10 copies/µL across all targets. no cross-reactivity against non-target organisms, and cycle threshold (Ct) values below 30 for positive controls. In clinical reference panels, the assay achieved 98.2% positive agreement and 100% negative agreement for *S. haematobium*, and 100% sensitivity and specificity for all hr-HPV genotypes. In a set of 217 cervical samples, the multiplex format produced results consistent with those obtained in single-plex reactions. Two additional HPV 45-positive samples were detected in the multiplex assay, likely reflecting low-level target detection near the assay’s sensitivity threshold and minor variation between single-plex and multiplex reaction conditions. Concordance with reference methods was high: 93.6% (κ = 0.87) compared with visual inspection/microscopy for female genital schistosomiasis (FGS), and 90.3–99.5% (κ = 0.80–0.95) compared with the ScreenFire assay for hr-HPV. These findings support the performance of the assay for simultaneous detection and highlight its potential to fill diagnostic gaps in endemic regions, where FGS-associated inflammation may contribute to HPV persistence and cervical carcinogenesis [6,9,26,27].

Key strengths of the assay include high sensitivity at low DNA levels, no amplification was observed in the non-target organisms included in the study panel, and the assay showed consistent performance in multiplex format without loss of detection efficiency. Traditional diagnostic methods for FGS, including visual inspection and microscopy, rely on observing tissue changes or parasitological evidence of eggs or lesions, and are subjective and operator-dependent. Urine microscopy and visual cervical inspection are imperfect reference standards with documented modest sensitivity; therefore, the reported metrics reflect agreement with these comparators rather than true clinical sensitivity and specificity against a perfect gold standard [13,28,29]. Microscopy of cervical or urinary specimens has reported sensitivities of 50–70%, limited by intermittent egg shedding, while VIA achieves 65–78% sensitivity and 75–96% specificity for FGS-associated dysplasia but misses subclinical disease [13,28,29]. In the present validation, the qPCR assay detected 11 additional *S. haematobium* positives that were negative by visual assessment/microscopy, likely reflecting detection of low-burden or egg-negative infections via DNA amplification. The detection of additional PCR-positive cases aligns with prior studies showing molecular methods identify low-burden infections missed by microscopy. Concordant findings have been reported in studies from Zanzibar and Kenya showing that PCR frequently detects more infections than microscopy by capturing low-level or sub-patent infections [20,29]. Thus, targeting the Dra1 repeat provides an objective, quantifiable readout, and therefore represents a useful complement to existing methods in confirmatory or high-risk settings.

The developed assay also demonstrated excellent specificity, with no amplification observed for common uropathogenic bacteria, including *E. coli, K. pneumoniae*, *S. aureus,* and non-target HPV genotypes, and *S. mansoni*. The selection of seven HPV genotypes aligns with WHO target product profiles prioritizing high specificity for clinically relevant types [30]. Although additional HPV types could be included, most contribute minimally to cervical cancer, and expanding panels risks reduced specificity. Further studies will consider the possibility of including HPV 35, common in sub-Saharan Africa [31,32].

The multiplex design supports integrated screening for FGS and hr-HPV, in line with WHO recommendations for combining diagnostics for cervical cancer prevention and neglected tropical diseases [17]. Simultaneous detection from a single cervical swab can streamline clinical workflows, reduce patient visits, and facilitate earlier management in co-endemic regions. Applications include integration into STI services, cervical cancer prevention programs, or targeted screening of high-risk populations [3,33]. While the current open-platform multiplex design offers practical advantages for integrated FGS and hr-HPV detection in resource-limited laboratories equipped with standard real-time PCR instruments, routine deployment should incorporate a process internal control to monitor extraction and inhibition, as recommended by CLSI guidelines [34]. The foundational performance data reported here provide a strong basis for such next-step optimization.

The multiplex qPCR assay represents a promising molecular approach for the combined detection of *S. haematobium* and high-risk HPV. Its open real-time PCR platform is compatible with standard real-time thermocyclers, enabling use in laboratories without dependence on proprietary cartridges. The assay has potential to strengthen early detection, improve diagnostic integration, and support public health strategies in settings where both FGS and HPV-related diseases remain major health challenges.

The feasibility of deploying this assay in lower-level healthcare facilities or field settings remains to be determined and will depend on infrastructure, cost, and technical capacity. These factors may influence its potential public health impact, particularly in resource-limited and endemic regions.

### Limitations and public health implications

This study has several limitations. All evaluation panels were derived from archived specimens, which, while well-characterized and previously tested, may not fully reflect pre-analytical variability encountered during routine specimen collection, transport, and storage in prospective settings. Second, although reactions were run in triplicate with nuclease-free water negative controls and non-template controls (NTCs) to monitor contamination and reaction validity, a dedicated internal control (exogenous process control or endogenous human target) was not incorporated to assess DNA extraction efficiency and potential PCR inhibition on a per-sample basis. For the analytical validation component, purified reference plasmids and biobanked DNA were used, minimizing extraction-related concerns. For the 217 clinical cervical swabs, DNA was freshly extracted using a standardized kit and quantified fluorometrically prior to amplification; performance remained consistent with single-plex results and showed strong agreement with independent reference methods (κ = 0.80–0.95). No evidence of systematic inhibition (e.g., unexpectedly high Ct values or failed runs) was observed. Nevertheless, the absence of a formal extraction/process internal control represents an important methodological consideration for multiplex assays, where target competition could theoretically exacerbate inhibition effects in complex clinical matrices. We acknowledge this gap and note that any future implementation or prospective validation studies will incorporate a suitable internal control with corresponding multiplex re-optimization. Nevertheless, our approach aligns with how similar pragmatic multiplex (or qPCR) assays for NTDs/helminths and HPV have been reported in the literature [2–4], which routinely employ phased analytical and clinical performance evaluation on characterized panels from endemic or high-risk cohorts [19,35]. Third, visual identification of FGS-like lesions is subject to inter-observer variability, which may influence clinical classification. Additionally, the pooling of certain HPV genotypes in the multiplex assay, while improving efficiency, may require follow-up testing for genotype resolution. Despite these limitations, the developed multiplex qPCR assay demonstrates potential utility for integrated detection of *S. haematobium* infection and high-risk HPV. Such an approach could enhance screening efficiency and reduce diagnostic costs in endemic and resource-limited settings, thereby supporting early detection and improved management of co-infections that may contribute to cervical disease burden.

## Conclusion

This study describes the development and validation of a qPCR assay for the detection of *S. haematobium* and seven high-risk HPV genotypes. The assay demonstrated consistent analytical performance and agreement with established diagnostic methods under the conditions evaluated. These findings suggest that the assay may serve as a useful tool for the combined detection of *S. haematobium* infection and HPV, particularly in settings with access to qPCR platforms. Further studies are needed to evaluate its performance in broader populations and routine clinical settings.

## Supporting information

S1 Appendix Additional result tables. (DOCX)

S2 Appendix Additional figures. (DOCX)

## Abbreviations

CeRAB: Centre for Research in Applied Biology
CCVP: CERVIVAL Clinical Validation Panel
CI: confidence interval
Ct: cycle threshold
FGS: Female Genital Schistosomiasis
ARP: Analytical Reference Panel
HCRP: HPV Clinical Reference Panel
HPV: Human papillomavirus
hr-HPV: high-risk Human papillomavirus
IHRC: International Human Papillomavirus Reference Center
LoD: Limit of detection
NPV: Negative predictive value
NTC/NTCs: non-template control(s)
PBS: phosphate-buffered saline
PPV: Positive predictive value
qPCR: quantitative real-time polymerase chain reaction
SCRP: *S. haematobium* Clinical Reference Panel
STI: sexually transmitted infection
VI: visual inspection
VIA: Visual inspection with acetic acid
WHO: World Health Organization
