# Supplementary material for: Development of a multiplex quantitative real-time PCR (qPCR) assay for simultaneous detection of Schistosoma haematobium and high-risk human papillomaviruses
Source: PLoS Negl Trop Dis. 2026 Sep 11;20(9):e0014694. doi: 10.1371/journal.pntd.0014694 (PMC13592694; doi:10.1371/journal.pntd.0014694)
Supplement: Supplementary file 1 [file pntd.0014694.s001.docx]

Table A in S1 Appendix: Overview of validation panels used in the study

| **Panel Identifier** | **Sample type** | **n** | **Positive / Negative definition (reference method)** | **Purpose** |
| --- | --- | --- | --- | --- |
| ARP | Plasmids + biobanked *S. haematobium* DNA | — | Defined by known plasmid concentration / known positive DNA | Analytical validation (specificity, LoD) |
| SCRP | Biobanked urine-derived DNA | 94 | Urine microscopy (56 positive, 38 negative) | Clinical sensitivity/specificity – *S. haematobium* |
| HCRP | Extracted cervical DNA | 132 | ScreenFire HPV assay (87 positive, 45 negative) | Clinical sensitivity/specificity – hr-HPV |
| CCVP | Archived cervical swabs | 217 | VIA + urine microscopy + ScreenFire HPV assay | Multiplex clinical performance & agreement |

Note: ARP: Analytical Reference Panel; CCVP: CERVIVAL Clinical Validation Panel; HCRP: HPV Clinical Reference Panel; SCRP: *S.* haematobium Clinical Reference Panel

Table B in S1 Appendix: Analytical Specificity of the developed qPCR assay for detecting S. haematobium and HPV genotypes

| Organisms | Cycle threshold observed |
| --- | --- |
| *Escherichia coli* | Undetected |
| *Klebsiella pneumoniae* | Undetected |
| *Staphylococcus aureus* | Undetected |
| HPV16 (tested with HPV 18 assay) | Undetected |
| HPV 18 (tested with HPV 45assay) | Undetected |
| *Schistosoma mansoni* | Undetected |

Table C in S1 Appendix. Analytical sensitivity (or LOD)

| *Sample Concentration(copy/µL)* | 1.0× 10^7^ | 1.0× 10⁶ | 1.0× 10⁵ | 1.0× 10⁴ | 1.00× 10^3^ | 1.0× 10^2^ | 1.0× 10¹ | 1.0× 10⁰ |
| --- | --- | --- | --- | --- | --- | --- | --- | --- |
| *Genotype-Specific Positive Rates (%)* | | | | | | | | |
| *S. haematobium* | 100 | 100 | 100 | 100 | 100 | 100 | 95 | 95 |
| HPV 16 | 100 | 100 | 100 | 100 | 100 | 100 | 100 | 90 |
| HPV 18 | 100 | 100 | 100 | 100 | 100 | 100 | 95 | 75 |
| HPV 31 | 100 | 100 | 100 | 100 | 100 | 95 | 95 | 50 |
| HPV 33 | 100 | 100 | 100 | 100 | 100 | 100 | 100 | 80 |
| HPV 45 | 100 | 100 | 100 | 100 | 100 | 95 | 95 | 45 |
| HPV 52 | 100 | 100 | 100 | 100 | 100 | 100 | 95 | 65 |
| HPV 58 | 100 | 100 | 100 | 100 | 100 | 100 | 100 | 90 |

The lowest concentration at which the assay yielded a positive result for all target strains when tested in replicate (20 replicates) was 10 copies/ µL.

Table D in S1 Appendix: Cycle threshold values observed in the single-plex qPCR assay for 217 clinical samples

| Target gene | % Positive | Ct Min | Ct Max | Mean Ct ± SD |
| --- | --- | --- | --- | --- |
| Dra1 | 123(56.7) | 15.72 | 34.81 | 27.07±5.94 |
| HPV 16 E6/E7 | 32(14.7) | 20.76 | 32.22 | 25.42±3.33 |
| HPV 18 E6/E7 | 19(8.8) | 20.94 | 31.99 | 25.86±3.27 |
| HPV 45 E6/E7 | 9(4.10 | 21.77 | 27.95 | 25.20±2.28 |
| HPV 31 E6/E7 | 14(6.5) | 22.67 | 30.10 | 26.10±2.36 |
| HPV 33 E6/E7 | 21(9.7) | 19.92 | 32.14 | 24.50±3.61 |
| HPV 52 E6/E7 | 27(12.4) | 20.18 | 32.88 | 25.11±3.54 |
| HPV 58 E6/E7 | 43(19.8) | 19.59 | 31.88 | 24.86±3.22 |

Table E in S1 Appendix: Clinical sensitivity and specificity for S. haematobium

| Parameters | Value (%) | 95% confidence interval |
| --- | --- | --- |
| Clinical Sensitivity | 98.2 | 90.7 – 99.9 |
| Clinical Specificity | 100.0 | 90.8 – 100.0 |
| PPV | 100.0 | 93.5 – 100.0 |
| NPV | 97.4 | 86.2 – 99.5 |

PPV: Positive predictive value; NPV: Negative predictive value: 95% Confidence Interval (CI)

Table F in S1 Appendix: Clinical sensitivity and specificity for HPV genotypes

| Parameters | Value (%) | 95% confidence interval |
| --- | --- | --- |
| Clinical Sensitivity | 100 | 95.8 – 100 |
| Clinical Specificity | 100 | 92.1 – 100 |
| PPV | 100 | 95.8 – 100 |
| NPV | 100 | 92.1 – 100 |

PPV: Positive predictive value; NPV: Negative predictive value: 95% Confidence Interval (CI)

Table G in S1 Appendix: Optimized Primer and Probe Concentrations for Multiplex qPCRs

| Target | Primer Con. (nM) | Primer Volume (µL, 10 µM stock) | Probe Con. (nM) | Probe Volume (µL, 10 µM stock) |
| --- | --- | --- | --- | --- |
| *S. haematobium* (Dra1) | 300 | 0.6 µL | 200 | 0.4 |
| HPV16 | 250 | 0.5 µL | 200 | 0.4 |
| HPV18 | 250 | 0.5 µL | 200 | 0.4 |
| HPV31 | 300 | 0.6 µL | 250 | 0.5 |
| HPV33 | 300 | 0.6 µL | 250 | 0.5 |
| HPV45 | 250 | 0.5 µL | 200 | 0.4 |
| HPV52 | 350 | 0.7 µL | 250 | 0.5 |
| HPV58 | 350 | 0.7 µL | 250 | 0.5 |
