# Supplementary material for: Development of a multiplex quantitative real-time PCR (qPCR) assay for simultaneous detection of Schistosoma haematobium and high-risk human papillomaviruses
Source: PLoS Negl Trop Dis. 2026 Sep 11;20(9):e0014694. doi: 10.1371/journal.pntd.0014694 (PMC13592694; doi:10.1371/journal.pntd.0014694)
Supplement: Supplementary file 2 [file pntd.0014694.s002.docx]

Fig A in S2 Appendix. Single-plex qPCR for the developed primer and probe

Fig B in S2 Appendix: Fluorescence amplification curve of qPCR reaction

Fig C in S2 Appendix. Multiplex qPCR for the developed assay
